# The combination of diethyldithiocarbamate and copper ions is active against *Staphylococcus aureus* and *Staphylococcus epidermidis* biofilms *in vitro* and *in vivo*

**DOI:** 10.3389/fmicb.2022.999893

**Published:** 2022-09-09

**Authors:** Laurine Kaul, Adrian I. Abdo, Tom Coenye, Bastiaan P. Krom, Michel A. Hoogenkamp, Andrew C. W. Zannettino, Regine Süss, Katharina Richter

**Affiliations:** ^1^Richter Lab, Basil Hetzel Institute for Translational Health Research, Department of Surgery, University of Adelaide, Adelaide, SA, Australia; ^2^Department of Pharmaceutical Technology and Biopharmacy, Institute of Pharmaceutical Sciences, University of Freiburg, Freiburg, Germany; ^3^Faculty of Health and Medical Sciences, Adelaide Medical School, University of Adelaide, Adelaide, SA, Australia; ^4^Laboratory of Pharmaceutical Microbiology, Ghent University, Gent, Belgium; ^5^Department of Preventive Dentistry, Academic Center for Dentistry Amsterdam (ACTA), University of Amsterdam and Vrije Universiteit Amsterdam, Amsterdam, Netherlands; ^6^Precision Medicine Theme, South Australian Health & Medical Research Institute, Adelaide, SA, Australia; ^7^Central Adelaide Local Health Network, Adelaide, SA, Australia; ^8^Institute for Photonics and Advanced Sensing, University of Adelaide, Adelaide, SA, Australia

**Keywords:** biofilm, antibacterial, diethyldithiocarbamate, copper ions, *Staphylococcus aureus*, *Staphylococcus epidermidis*, new treatment

## Abstract

*Staphylococcus aureus* and *Staphylococcus epidermidis* are associated with life-threatening infections. Despite the best medical care, these infections frequently occur due to antibiotic resistance and the formation of biofilms of these two bacteria (i.e., clusters of bacteria embedded in a matrix). As a consequence, there is an urgent need for effective anti-biofilm treatments. Here, we describe the antibacterial properties of a combination treatment of diethyldithiocarbamate (DDC) and copper ions (Cu^2+^) and their low toxicity *in vitro* and *in vivo*. The antibacterial activity of DDC and Cu^2+^ was assessed *in vitro* against both planktonic and biofilm cultures of *S. aureus* and *S. epidermidis* using viability assays, microscopy, and attachment assays. Cytotoxicity of DDC and Cu^2+^ (DDC-Cu^2+^) was determined using a human fibroblast cell line. *In vivo* antimicrobial activity and toxicity were monitored in *Galleria mellonella* larvae. DDC-Cu^2+^ concentrations of 8 μg/ml DDC and 32 μg/ml Cu^2+^ resulted in over 80% MRSA and *S. epidermidis* biofilm killing, showed synergistic and additive effects in both planktonic and biofilm cultures of *S. aureus* and *S. epidermidis*, and synergized multiple antibiotics. DDC-Cu^2+^ inhibited MRSA and *S. epidermidis* attachment and biofilm formation in the xCELLigence and Bioflux systems. *In vitro* and *in vivo* toxicity of DDC, Cu^2+^ and DDC-Cu^2+^ resulted in > 70% fibroblast viability and > 90% *G. mellonella* survival. Treatment with DDC-Cu^2+^ significantly increased the survival of infected larvae (87% survival of infected, treated larvae *vs*. 47% survival of infected, untreated larvae, *p* < 0.001). Therefore, DDC-Cu^2+^ is a promising new antimicrobial with activity against planktonic and biofilm cultures of *S. epidermidis* and *S. aureus* and low cytotoxicity *in vitro*. This gives us high confidence to progress to mammalian animal studies, testing the antimicrobial efficacy and safety of DDC-Cu^2+^.

## Introduction

The Gram-positive bacteria *Staphylococcus aureus* and *Staphylococcus epidermidis* are notable human pathogens, causing infections ranging from mild skin infection to life-threatening bacteremia (Kleinschmidt et al., 2015; López-Cortés et al., 2020), endocarditis (Cahill et al., 2017), osteoarticular (Kaushik and Kest, 2018) and medical device related infections (Otto, 2008; Zheng et al., 2018; Patiniott et al., 2022). Furthermore, *S. aureus* is the most common pathogen isolated from surgical site infections (Tong et al., 2015). Typically, a bacterial infection is treated with antibiotics (Stevens et al., 2005), e.g., intervention against *S. aureus* infections is executed with either β-lactams, lincosamides, lipopeptides, tetracyclines, glycopeptides, linezolid, or adjunct trimethoprim-sulfamethoxazole therapy (Liu et al., 2011). However, these therapies are frequently failing due to the rise of antibiotic resistance and the formation of biofilms (Santajit and Indrawattana, 2016).

Biofilms are aggregates of bacteria embedded in a protective matrix (Costerton et al., 1999) and are known to be up to 1,000-fold more tolerant to antimicrobial agents compared to planktonic cells (Mah and O’Toole, 2001). The biofilm matrix, a conglomeration of extracellular polymeric substances, prevents diffusion of the drug and modulates or reduces their metabolic activity (Crabbé et al., 2019). In addition, staphylococci developed penicillin-resistance, including methicillin-resistant *S. aureus* (MRSA) with rates varying between 1.5 and over 50% in different parts of the world (Australian Commission on Safety and Quality in Health Care (ACSQHC), 2019; Craft et al., 2019; European Centre for Disease Prevention and Control, 2022) and methicillin-resistant *S. epidermidis* with reported rates over 70% (Lee et al., 2018). The implications of antimicrobial resistance are devastating, as exemplified by MRSA-associated surgical site infections, which is associated with 2- to 11-fold increased patient mortality (Anderson et al., 2007). Therefore, *S. aureus* is listed as a high priority pathogen for research and development by the World Health Organization, emphasizing the urgency for new treatments (World Health Organization, 2017).

Innovative strategies against *S. aureus* and *S. epidermidis* in the research and development pipeline include newly synthesized compounds (Dinarvand et al., 2020; Sovari et al., 2020; Wang et al., 2021), bacteriophages (Feng et al., 2021; Walsh et al., 2021), metals (Richter et al., 2017a; Sánchez-López et al., 2020) and repurposed drugs (Thangamani et al., 2015; Richter, 2019). Repurposing of drugs has a history of multiple benefits and safe uses, allowing for a faster bench to bedside translation and lower drug development costs (Pushpakom et al., 2018).

An excellent candidate for drug repurposing is diethyldithiocarbamate (DDC). DDC is the metabolite of disulfiram, an FDA-approved drug for the treatment of chronic alcoholism, which have both recently resurfaced as potentially useful in other medical fields, such as cancer, cocaine addiction, or infections with fungi, parasites, viruses and bacteria (Kaul et al., 2021). DDC showed high antifungal activity against *Candida albicans* and *Candida tropicalis* biofilms (Harrison et al., 2007), reduced the load of *Leishmania braziliensis* (Khouri et al., 2010; Celes et al., 2016) and, in combination with copper ions (Cu^2+^), showed anti-SARS-CoV-2 activity by targeted oxidation strategies (Xu et al., 2021). The suggested mechanisms behind the antimicrobial activity of DDC is based on chelating vital metals and inhibiting enzymes (Phillips et al., 1991), such as the carbonic anhydrases present in *Legionella pneumophila* (Nishimori et al., 2014) or the superoxide dismutase present in *Candida albicans* (De Brucker et al., 2013), *Leishmania braziliensis* (Khouri et al., 2010) or *Bacillus anthracis* (Frazier et al., 2019). An additional advantage of DDC is a lack of teratogenic, mutagenic or carcinogenic effects in animal models (Gessner and Gessner, 1992).

Based on the anti-cancer activity of DDC being linked to the addition of Cu^2+^ and on limited activity against Gram-positive bacteria of DDC as monotherapy, DDC was combined with copper ions (Cu^2+^) and showed promising results against mycobacteria and streptococci (Dalecki et al., 2015; Menghani et al., 2021). However, the combination of DDC with metal ions, such as Cu^2+^ has not been further investigated against staphylococci and their biofilms. Thus, this study presents the antibacterial activity of DDC and Cu^2+^ against planktonic and biofilm *S. aureus* and *S. epidermidis* including *in vivo* safety and efficacy in an infected *Galleria mellonella* model.

## Materials and methods

### Bacterial strains and cell cultures

*Staphylococcus epidermidis* ATCC 35984 and ATCC 14990, *S. aureus* ATCC 25923 and ATCC 700699 (also known as MRSA Mu50), and *Escherichia coli* ATCC 25922 were purchased from the American Type Culture Collection (Manassas, VA, United States). Three clinical isolates, i.e., MRSA 1, 2 and 3 were obtained from Adelaide Pathology Partners (Mile End, Australia). *Pseudomonas aeruginosa* PAO1 was obtained from the School of Molecular Medical Sciences, University of Nottingham (Nottingham, United Kingdom). Unless stated otherwise, bacterial suspensions were prepared by dissolving colonies in 0.9% saline and adjusted to the appropriate McFarland units before being further diluted in broth and incubated at 37°C under aerobic conditions. Cell culture studies were carried out using control human fibroblast cells (Coriell Cat# GM00038, RRID: CVCL_7271) obtained from the Coriell Institute for Medical Research (Camden, NJ, United States). Unless stated otherwise, all experiments were carried out at least in triplicate and all chemicals, media and supplements were purchased from Sigma-Aldrich (Steinheim, Germany).

### Minimal inhibitory concentration and checkerboard analysis

The MIC values of DDC (Carl Roth, Karlsruhe, Germany) and the antibiotics methicillin (Meth), ceftazidime (Ceft), vancomycin (Van), ciprofloxacin (Cip), doxycycline (Doxy), amikacin (Amik) and erythromycin (Erythro) towards the staphylococci *S. aureus* and *S. epidermidis* and the Gram-negative bacteria *E.* coli and *P. aeruginosa* were determined in a 96-well microtiter plate using the broth microdilution method (Wiegand et al., 2008). Bacterial suspensions were adjusted to 0.5 ± 0.1 McFarland units, further 1: 100 diluted in Mueller-Hinton broth (Thermo Fisher) and mixed with equal volumes of treatments or antibiotics. Treatment concentrations of DDC ranged from 0.5 to 128 μg/ml and for antibiotics from 0.125 to 64 μg/ml. Furthermore, the broth microdilution method was adapted to investigate the MIC of gallium nitrate hydrate (Ga^3+^), iron sulphate heptahydrate (Fe^2+^), calcium chloride dihydrate (Ca^2+^), magnesium sulphate (Mg^2+^), zinc sulphate heptahydrate (Zn^2+^) and copper sulphate pentahydrate (Cu^2+^) alone or in combination with DDC. The MIC was determined as the lowest concentration of treatment required to inhibit visual growth by the unaided eye (Wiegand et al., 2008).

### Biofilm checkerboard assay

Black 96-well microtiter plates (Costar, Corning Incorporated, NY, United States) were inoculated with 100 μl of a 1: 100 diluted *S. aureus*, MRSA or *S. epidermidis* bacterial suspension in nutrient broth, adjusted to 0.5 ± 0.1 McFarland units, and incubated at 37°C for 24 h on a rotating platform at 70 rpm (3D Gyratory Mixer, Ratek Instruments, Boronia, Australia). After washing once with sterile 0.9% w/v saline to remove planktonic bacteria, biofilms were exposed to serial dilutions of (i) 1 to 256 μg/ml DDC, (ii) 4 to 256 μg/ml Cu^2+^, (iii) mixture of DDC and Cu^2+^, (iv) antibiotics with concentrations ranging from 0.5 to 128 μg/ml, including Meth, Ceft, Van, Cip, Doxy, Amik and Erythro or (v) a mixture of DDC-Cu^2+^ and antibiotics, and further incubated at 37°C on a rotating platform for 24 h. After a second washing step to remove the treatments, bacterial viability was assessed by the AlamarBlue cell viability assay (Peeters et al., 2008; Richter et al., 2016). Briefly, 100 μl of a freshly prepared 10% v/v AlamarBlue (Thermo Fisher, MA, United States) solution in nutrient broth (Thermo Fisher) were added to each well and incubated, protected from light, for up to 5 h at 37°C on a rotating platform. The fluorescence was determined hourly using a FLUOstar OPTIMA plate reader (BMG LABTECH, Offenburg, Germany) at λ_excitation_/λ_emission_ = 530/590 nm. After reaching maximum fluorescence the relative biofilm killing efficacy was quantified according to Equation 1.


(1) $$\%\;\mathrm{Biofilm killing}=\left(1-\frac{I_{\mathrm{treatment}}-I_{\mathrm{blank}}}{I_{\mathrm{untreated}}-I_{\mathrm{blank}}}\right)\times 100$$

Antibiofilm activity of the different treatments was determined as percentage of biofilm killing, where the fluorescence intensity of treated and untreated biofilms is represented by I_treatment_ and I_untreated_, respectively, and I_blank_ represents the background fluorescence of the 10% v/v AlamarBlue solution (Richter et al., 2016).

### Synergy of compounds

The fractional inhibitory concentration index (FICi) was used to describe synergistic, additive, and antagonistic effects between DDC and Cu^2+^, or between DDC-Cu^2+^ and antibiotics. The equation for calculating the sum of FICi (ΣFICi) is based on the planktonic and biofilm checkerboard assay and exemplified for planktonic bacteria in Equation 2 using the MICs.


(2) $$\sum \mathrm{FICi}=\frac{{\mathrm{MIC}}_{ab}}{{\mathrm{MIC}}_{a}}+\frac{{\mathrm{MIC}}_{ba}}{{\mathrm{MIC}}_{b}}$$

MIC_*ab*_ = MIC of compound a in combination with b; MIC_*a*_ = MIC of compound a; MIC_*ba*_ = MIC of compound b in combination with a; MIC_*b*_ = MIC of compound b (Khouri et al., 2010). Similarly, the equation for biofilms was adapted by replacing the MIC with the minimum biofilm inhibitory concentration, correlating to a minimum of 80% biofilm killing. According to previous literature, the ΣFICi was interpreted as: (i) synergy; ΣFICi ≤0.5, (ii) additivity; ΣFICi between 0.5 and 1, (iii) indifference; ΣFICi ≥1 and ≤4, and (iv) antagonism; ΣFICi ≥4 (Sopirala et al., 2010).

### Confocal microscopy

An 8-well chamber slide (μ-Slide, Ibidi, Gräfelfing, Germany) was inoculated with 300 μl of a 1: 100 dilution of a bacterial suspension of MRSA Mu50 or *S. epidermidis* ATCC 35984 adjusted to 0.5 ± 0.1 McFarland units in nutrient broth and incubated for 24 h at 37°C on a rotating platform at 70 rpm (3D Gyratory Mixer, Ratek Instruments, Boronia, Australia). Biofilms were rinsed with phosphate buffered saline, followed by exposure to DDC-Cu^2+^ (8 μg/ml DDC + 32 μg/ml Cu^2+^) or nutrient broth alone for 24 h at 37°C on a rotating platform. After a second washing step, a 1: 1000 dilution of LIVE/DEAD BacLight staining (SYTO 9/propidium iodide; Life Technologies, Scoresby, Australia) was incubated in the dark for 30 min, then imaged by confocal laser scanning microscopy (Olympus FV3000, Shinjuku, Japan) using a 20 × and 100 × objective. The excitation/emission wavelengths of the LIVE/DEAD BacLight staining were 488/520 nm and 543/619 nm, respectively. The images were quantified using ImageJ Software (1.53q, NIH, University of Wisconsin, WI, United States). Due to the number of layers of cells in the biofilm and the magnification objective, live/dead cell count was not possible. Instead, measurement of total red and green fluorescence ratio was used to semi-quantitatively calculate the live/dead cell ratio.

### Prevention of bacterial attachment

The activity of DDC-Cu^2+^ to inhibit bacterial attachment was determined using the xCELLigence real-time cell analysis (RTCA; Agilent, CA, United States). This technology measures the impedance through gold electrode sensors placed on the bottom of each well of the RTCA E-plate 16 (Agilent, CA, United States). When cells attach onto the electrodes, a larger impedance is detected, leading to an increase of the cell index (CI) compared to the baselines.

To measure the baselines, 50 μl of nutrient broth and 100 μl of 8 μg/ml DDC, 32 μg/ml Cu^2+^, DDC-Cu^2+^ (8 μg/ml DDC + 32 μg/ml Cu^2+^) dissolved in nutrient broth or media alone were added to each well. A bacterial overnight culture in nutrient broth was adjusted to OD_600_ of 0.4 for MRSA Mu50 and *S. epidermidis* ATCC 35984. A 1: 4 dilution of the bacterial suspension was added to the appropriate wells. The impedance of the cells was continuously and automatically measured every 15 min for 48 h while statically incubated at 37°C. Wells with bacterial suspension in broth (100% bacterial attachment), wells with broth alone (background) and wells with compounds in broth (0% bacterial attachment, reflecting the compounds’ influence on impedance) were assessed as controls.

### Bioflux

The Bioflux system (Fluxion, United States) was used to determine inhibition of biofilm growth under flow conditions, as previously described (Hoogenkamp, 2021). All media was pre-warmed to 37°C before use. Bioflux plates were primed with 350 μl half-strength tryptone soy broth (TSB, BD, Sparks, MD, United States) and inoculated with 70 μl of a bacterial overnight culture (either MRSA Mu50 or *S. epidermidis* ATCC 35984) adjusted to OD_600_ of 0.2. Following bacterial attachment for 30 min at 37°C and no flow, bacteria were exposed to either half-strength TSB or half-strength TSB supplemented with DDC-Cu^2+^ (8 μg/ml DDC + 32 μg/ml Cu^2+^) for 24 h at 37°C under steady nutrient flow (0.5 dyne/cm^2^). Biofilm growth was monitored through brightfield microscopy (20× objective), and images were automatically taken every 15 min.

### *In vitro* cytotoxicity

The GM00038 normal human skin fibroblast cell line was cultured in Eagle’s Minimum Essential Medium with Earle’s salts and non-essential amino acids supplemented with 15% fetal bovine serum (Biochrom, Berlin, Germany) and 2.2 g/l sodium bicarbonate anhydrous. Fibroblasts were seeded at 5 × 10^4^ cells/100 μl culture medium per well in black 96-well flat-bottom plates and incubated at 37°C in 5% CO_2_ for 24 h to allow attachment. Cells were separately treated with either 8 μg/ml DDC, 32 μg/ml Cu^2+^ or DDC-Cu^2+^ (8 μg/ml DDC + 32 μg/ml Cu^2+^) for 18 h. The effect of the compounds on fibroblast viability was assessed with the CellTiter-Glo^®^ Luminescent Viability Assay (Promega Corporation, WI, United States) according to the manufacturer’s instructions and luminescence was measured on a FLUOstar OPTIMA plate reader. Equation 3 was used to quantify the percentage of fibroblast viability, where the luminescence intensity of treated and untreated fibroblast cells is represented by I_treatment_ and I_untreated_, respectively, and I_blank_ represents the background luminescence of the CellTiter-Glo® reagent.


(3) $$\%\;\mathrm{Fibroblast viability}=\left(\frac{I_{\mathrm{treatment}}-I_{\mathrm{blank}}}{I_{\mathrm{untreated}}-I_{\mathrm{blank}}}\right)\times 100$$

### *In vivo* cytotoxicity and efficacy

*Galleria mellonella* larvae (Hengelsport De Poorterwere, Ghent, Belgium) were stored in the dark at 13°C and used within 3 days of receipt. Each treatment group was assigned 30 larvae. Larvae were injected in the last proleg with micro-fine (30 gauge) needle insulin syringes (BD, Franklin Lakes, NJ, United States). Three control groups were included, (i) larvae injected with 0.9% saline (uninfected, untreated control), (ii) larvae injected with treatment (uninfected, treated control to determine treatment toxicity) and (iii) larvae injected with a bacterial suspension (infected, untreated control). To determine treatment efficacy, larvae were injected with a bacterial suspension (either MRSA Mu50 or *S. epidermidis* ATCC 35984) and with DDC, Cu^2+^or DDC-Cu^2+^. Considering the dilution factor within the larvae, the concentrations of the DDC-Cu^2+^ were increased a 10-fold and based on the average weight of the larvae (250 mg) was determined as 6.4 mg/kg DDC and 25.6 mg/kg. A total volume of 20 μl was injected comprising treatment or saline in a 1:1 mix with a bacterial suspension in nutrient broth. The final bacterial density was OD_600_ 0.05. Larvae were housed in petri dishes in the dark at 37°C and the larvae mortality was monitored daily over 4 days.

### Statistical analysis

Results were statistically analyzed using GraphPad Prism (RRID:SCR_002798) version 9.00 for Windows (GraphPad Software, CA, United States) and statistical significance was determined with an α = 0.05. Parametric data (MIC, biofilm killing and cytotoxicity) are represented by the mean ± standard deviation (SD), which was analyzed using one-way analysis of variance (ANOVA) with Dunnett’s (for MICs, biofilm checkerboard, microscopy) or Tukey’s (for xCELLigence) multiple comparison test for finding statistical differences between treatment groups. *G. mellonella* survival data was analyzed using Kaplan–Meier survival curves with significant differences between groups determined by log-rank test, significance was Bonferroni-Holm-corrected for multiple comparisons.

## Results

### Minimal inhibitory concentration

As shown in Table 1, DDC displayed low antibacterial activity against *S. epidermidis* ATCC 35984 with a MIC of 64 μg/ml. To increase the antibacterial activity of DDC, a selection of metal salts was evaluated against *S. epidermidis* ATCC 35984 in the presence or absence of DDC. The MIC of the metal salts alone was 128 μg/ml for Cu^2+^ and above 128 μg/ml for all other metal ions (Table 1). In combination with Ga^3+^, Fe^2+^ and Ca^2+^, the MIC of DDC was not reduced. In contrast, the MIC of DDC was reduced to 16 μg/ml in the presence of Mg^2+^ and Zn^2+^ and to 1 μg/ml when combined with Cu^2+^ (Table 1).

**Table 1:** Minimum inhibitory concentration (MIC) of diethyldithiocarbamate (DDC), metal ions and the combination of both against *S. epidermidis* ATCC 35984.

MIC (μg/ml)			
Metal ion	DDC	Metal ion	DDC^a^-Metal ion^b^
	64		
Ga^3+^		>128	64/>128
Ca^2+^		>128	64/>128
Fe^2+^		>128	32/4
Mg^2+^		>128	16/4
Zn^2+^		>128	16/4
Cu^2+^		128	1/8

a MIC of DDC in combination with metal ion.

b MIC of metal ion in combination with DDC.

Since the DDC combination with Cu^2+^ resulted in a substantial MIC reduction in *S. epidermidis* ATCC 35984, the MIC of DDC in the presence or absence of Cu^2+^ was further investigated in a range of bacteria. In *S. aureus*, MRSA and *S. epidermidis*, the MICs of DDC ranged from 32 to 128 μg/ml. The MIC of DDC against *E. coli* and *P. aeruginosa* was above 128 μg/ml. The extensive MIC reduction of DDC in the presence of Cu^2+^ was also observed with other *S. aureus*, MRSA and *S. epidermidis* strains (Table 2). Both the MIC of DDC in the presence of Cu^2+^ and the MIC of Cu^2+^ in the presence of DDC were reduced in all *S. aureus* and *S. epidermidis* strains tested. Interestingly, the MIC values of the combination were the highest with 4 μg/ml DDC and 64 μg/ml Cu^2+^ in *S. aureus* ATCC 25923, the most antibiotic susceptible strain, while the MIC values of the combination were lowest, with 0.5 μg/ml DDC and 2 μg/ml Cu^2+^ in MRSA 2 and MRSA Mu50, the strain with the highest antibiotic MICs. In all strains tested, the lowest concentration of Cu^2+^ required to inhibit *S. aureus* and *S. epidermidis* growth exceeded the lowest DDC concentration.

**Table 2:** Minimal inhibitory concentration of the antibiotics methicillin (Meth), ceftazidime (Ceft), ciprofloxacin (Cip), vancomycin (Van), doxycycline (Doxy), amikacin (Amik), erythromycin (Erythro) and the compounds diethyldithiocarbamate (DDC) and Cu^2+^ towards planktonic *S. aureus*, MRSA, *S. epidermidis*, *E. coli* and *P. aeruginosa.*

Bacterial strain	MIC (μg/ml)										Synergy	
	Meth	Ceft	Cip	Van	Doxy	Amik	Erythro	DDC	Cu^2+^	DDC^a^-Cu^2+^^b^	ΣFICi^c^	Result^d^
*S. aureus* ATCC 25923		32	0.25	1	≤0.125	8	0.5	32	>128	4/64	1.23	Indifferent
MRSA Mu50	>64	>64	16	2	4	32	>64	64	>128	≤0.5/2	0.14^e^	Synergy
MRSA 1	2	32	0.25	1	≤0.125	8	0.25	128	>128	2/8	0.67	Additive
MRSA 2		>64	2	1	≤0.125	8	0.5	32	>128	≤0.5/2	0.19^e^	Synergy
MRSA 3	2	32	0.5	1	≤0.125	4	>64	128	>128	2/16	0.88	Additive
*S. epidermidis* ATCC 14990		8	≤0.125	1	≤0.125	0.5	≤0.125	32	>128	2/16	0.93	Additive
*S. epidermidis* ATCC 35984	64	64	≤0.125	1	≤0.125	8	>64	64	128	1/8	0.87	Additive
*E. coli* ATCC 25922								>128	>128	ND	ND	ND
*P. aeruginosa* PAO1								>128	>128	ND	ND	ND

Antibacterial activity and synergistic effects of the combination of both compounds (DDC-Cu^2+^) against planktonic *S. aureus*, MRSA and *S. epidermidis*.

a MIC of DDC in combination with Cu^2+^.

b MIC of Cu^2+^ in combination with DDC.

c Average of all calculated fractional inhibitory concentration index sums of DDC-Cu^2+^ (ΣFICi) (*n* = 3).

d Results: synergy ≤ 0.5; additivity > 0.5 to ≤ 1; indifferent > 1.

e ΣFICi values calculated with the lowest concentration of DDC in combination with Cu^2+^ measured (0.5 μg/ml) and not with MIC.ND, not determined.

### Effect of different DDC and Cu^2+^ concentrations on biofilms

**Figure 1:**
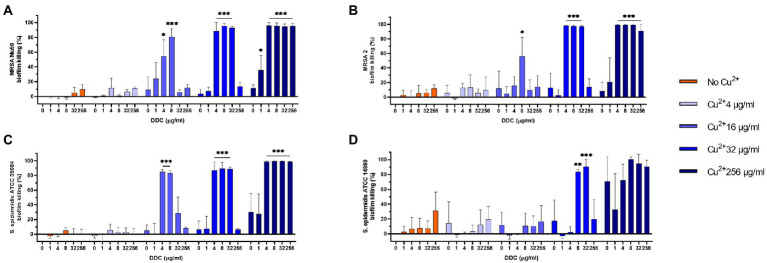
Effect of diethyldithiocarbamate (DDC) and Cu^2+^ concentrations (in μg/ml) on the viability of **(A)** MRSA Mu50, **(B)** MRSA 2, **(C)** *Staphylococcus epidermidis* ATCC 35984 and **(D)** *S. epidermidis* ATCC 14990 biofilms compared to monotherapy with Cu^2+^ (*n* = 3; ^*^p < 0.05; ^**^p < 0.01; ^***^p < 0.001).

MRSA and *S. epidermidis* biofilms were exposed to combined treatments of DDC (1 to 256 μg/ml) and Cu^2+^ (4 to 256 μg/ml). In Figure 1, the MRSA Mu50, MRSA 2, *S. epidermidis* ATCC 35984 and *S. epidermidis* ATCC 14990 biofilm killing of different DDC and Cu^2+^ combination (DDC-Cu^2+^) ratios were compared to the effect of single Cu^2+^ treatment. Overall, treatment with DDC alone, Cu^2+^ alone and combinations involving Cu^2+^ concentrations below 16 μg/ml resulted in low antibiofilm activity against *S. aureus* and *S. epidermidis* with less than 31.2% biofilm killing, except for Cu^2+^ 256 μg/ml against *S. epidermidis* ATCC 14990 resulting in 70.8% biofilm killing (Figure 1D). The highest biofilm killing was 95.8, 99.6, 99.3 and > 99.9% with 256 μg/ml Cu^2+^ in combination with 8 μg/ml DDC in MRSA Mu50 (Figure 1A), MRSA 2 (Figure 1B), *S. epidermidis* ATCC 35984 (Figure 1C) and *S. epidermidis* ATCC 14990 (Figure 1D), respectively. The minimal concentrations of DDC-Cu^2+^ that resulted in above 80.0% biofilm killing were 8 μg/ml DDC and 16 μg/ml Cu^2+^ in MRSA Mu50 (81.0% biofilm killing, *p* ≤ 0.001; Figure 1A), 4 μg/ml DDC and 32 μg/ml Cu^2+^ in MRSA 2 (98.6% biofilm killing, *p* ≤ 0.001, Figure 1B), 4 μg/ml DDC and 16 μg/ml Cu^2+^ in *S. epidermidis* ATCC 35984 (85.0% biofilm killing; *p* ≤ 0.001; Figure 1C) and 8 μg/ml DDC and 32 μg/ml Cu^2+^ in *S. epidermidis* ATCC 14990 (83.7% biofilm killing, *p* ≤ 0.01; Figure 1D). Complementing the results obtained against planktonic MRSA and *S. epidermidis*, low antibiofilm activity was observed when DDC concentrations exceeded Cu^2+^ concentrations, suggesting the importance of a DDC-Cu^2+^ ratio range. The lowest concentration of DDC and Cu^2+^ with over 80.0% biofilm killing in all strains tested was 8 μg/ml and 32 μg/ml Cu^2+^, therefore this concentration was chosen for further experiments. This concentration was also effective against *S. aureus* ATCC 25923, MRSA 1 and MRSA 3 biofilms (data not shown).

### Synergistic effects of DDC and Cu^2+^ in combination with different antibiotics

Synergistic and additive effects of DDC and Cu^2+^ were observed against all planktonic MRSA (ΣFICi: MRSA Mu50 = 0.14; MRSA 1 = 0.67; MRSA 2 = 0.19; MRSA 3 = 0.88) and *S. epidermidis* strains (ΣFICi: *S. epidermidis* ATCC 14990 = 0.93; *S. epidermidis* ATCC 35984 = 0.63), except for *S. aureus* ATCC 25923 (ΣFICi = 1.23; Table 2). Against the biofilm form of the same strains, the ΣFICi of DDC-Cu^2+^ was reduced in most strains (Table 3). Synergistic effects of the combination were reached against MRSA Mu50 (ΣFICi = 0.26), and additive effects were reached against both *S. epidermidis* strains (ΣFICi: *S. epidermidis* ATCC 14990 = 0.86; *S. epidermidis* ATCC 35984 = 0.58), *S. aureus* ATCC 25923 (ΣFICi = 0.80) and the other MRSA strains (ΣFICi: MRSA 1 = 0.53; MRSA 2 = 0.64; MRSA 3 = 0.66). The synergistic effects of DDC-Cu^2+^ in planktonic MRSA 2 and planktonic and biofilm MRSA Mu50 were not observed in the other MRSA strains tested, which showed additive effects of DDC-Cu^2+^. This difference should be investigated based on the phenotype and genotype of the different strains tested. As the MICs of multiple antibiotics were the highest for MRSA Mu50 and *S. epidermidis* ATCC 35984, respectively, these strains were chosen as representatives for *S. aureus* and *S. epidermidis* in the following experiments.

**Table 3:** Synergistic effects of diethyldithiocarbamate in combination with Cu^2+^ against *S. aureus,* MRSA and *S. epidermidis* biofilms.

Bacterial strain	Synergy	
	ΣFICi^a^	Results^b^
*S. aureus* ATCC 25923	0.80	Additive
MRSA Mu50	0.26	Synergy
MRSA 1	0.53	Additive
MRSA 2	0.64	Additive
MRSA 3	0.66	Additive
*S. epidermidis* ATCC 14990	0.86	Additive
*S. epidermidis* ATCC 35984	0.58	Additive

a Average of all calculated fractional inhibitory concentration index sums (ΣFICi) (*n* = 3).

b Results: synergy ≤ 0.5; additivity > 0.5 to ≤ 1; indifferent > 1.

The ΣFICi of the DDC-Cu^2+^ combination was further investigated with representatives of different classes of antibiotics against MRSA Mu50 biofilms (Table 4). The MRSA Mu50 strain was chosen based on the high antibiotics MICs in the planktonic form and on the biofilms not inhibited by antibiotics at concentrations of 128 μg/ml or lower, except for the tetracycline representative Doxy and the cell wall synthesis inhibitor Van (over 70% biofilm killing with concentrations of 16 μg/ml). When the antibiotics were combined with DDC-Cu^2+^, the minimum concentration to kill at least 80% of bacteria within the biofilm, was reduced at least 16-fold, except for the combination of Erythro with DDC-Cu^2+^ (no change). In addition, DDC-Cu^2+^ showed additive effect with Amik and the β-lactam antibiotics Meth and Ceft. Synergistic effects were observed when DDC-Cu^2+^ was combined with Cip, Doxy, and Van. However, no difference was observed with Erythro.

**Table 4:** Minimal concentration to kill over 80% biofilm and synergistic effects of antibiotics, diethyldithiocarbamate and Cu^2+^ (DDC-Cu^2+^) and the combination against MRSA Mu50 (*n* = 3).

Treatment	Minimal concentration to kill over 80% biofilm (μg/ml)			Synergy	
	Antibiotic	DDC-Cu^2+^	Antibiotic^a^/DDC-Cu^2+^^b^	ΣFICi^c^	Results^d^
DDC-Cu^2+^		4–16			
Meth	>128		8/0.5–2	0.63	Additive
Ceft	>128		8/0.5–2	0.71	Additive
Van	16		0.5/0.5–2	0.50	Synergy
Cip	>128		4/0.5–2	0.45	Synergy
Doxy	16		1/0.5–2	0.44	Synergy
Amik	>128		1/0.5–2	0.55	Additive
Erythro	>128		>128/4–16	1.43	Indifferent

a Lowest concentration of antibiotic in combination with DDC-Cu^2+^.

b Lowest concentration of DDC-Cu^2+^ in combination with antibiotic.

c Average of all calculated fractional inhibitory concentration index sums (ΣFICi) (*n* = 3).

d Results: synergy ≤ 0.5; additivity > 0.5 to ≤ 1; indifferent > 1.

### Visualizing biofilms after DDC-Cu^2+^ treatment

**Figure 2:**
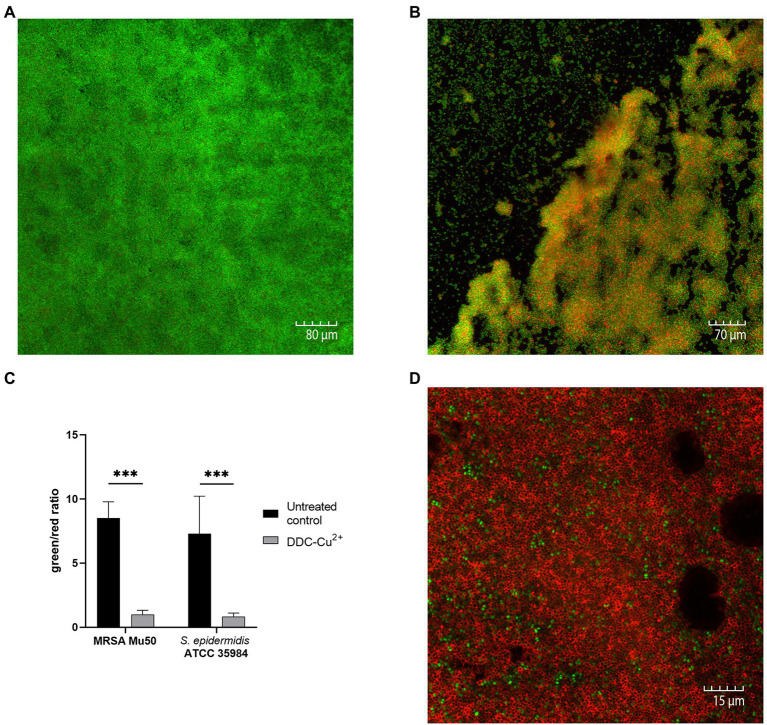
Comparison of stained MRSA Mu50 and *S. epidermidis* ATCC 35984 biofilms with LIVE/DEAD BacLight staining after treatment with 8 μg/ml diethyldithiocarbamate and 32 μg/ml Cu^2+^ (DDC-Cu^2+^). Confocal microscopy images results: green = viable bacteria; red = dead bacteria. **(A)** Untreated *S. epidermidis* ATCC 35984 biofilm at 20×. *S. epidermidis* ATCC 35984 biofilm after treatment with DDC-Cu^2+^ at **(B)** 20× and **(D)** 100×. **(C)** Quantification of images as green/red ratio of untreated control (black) and treatment with DDC-Cu^2+^ (grey) of MRSA and *S. epidermidis* ATCC 35984 biofilms (*n* = 3–8; ^***^p < 0.001).

Confocal microscopy images of the untreated control of *S. epidermidis* ATCC 35984 biofilms were characterized by a large, dense, and undisturbed biofilm with mostly viable bacteria (Figure 2A). After exposure to DDC-Cu^2+^ (8 μg/ml DDC + 32 μg/ml Cu^2+^), the biofilm structure was disturbed and less dense. In addition, an increase in number of red, indicating dead bacteria was observed (Figure 2B). Similar observations were made in MRSA Mu50 biofilm images (Supplementary Figure 1). The quantification of the fluorescence showed a significant decrease of the green/red ratio between untreated biofilm and biofilm treated with DDC-Cu^2+^ (Figure 2C). This ratio was also observed when using a 100 × objective on a DDC-Cu^2+^ treated *S. epidermidis* ATCC 35984 biofilm that showed dead bacteria with only few viable bacteria (Figure 2D).

### DDC-Cu^2+^ inhibits bacterial attachment

**Figure 3:**
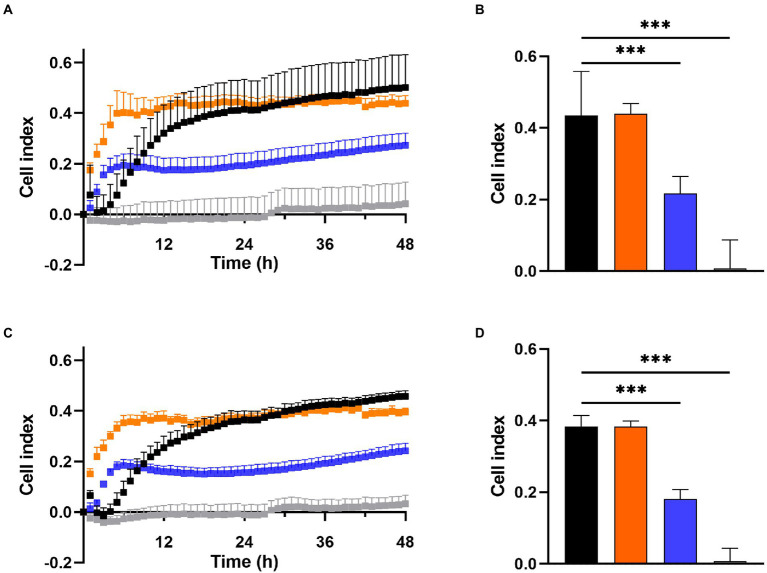
Effect of 8 μg/ml diethyldithiocarbamate (DDC; orange), 32 μg/ml Cu^2+^ (blue) and combined DDC-Cu^2+^ (grey) on **(A)** the cell index of MRSA Mu50 and **(C)** *S. epidermidis* ATCC 35984 over 48 h compared to the untreated control (black). Comparison of the mean cell index between 12 and 48 h for each treatment of **(B)** MRSA Mu50 and **(D)** *S. epidermidis* ATCC 35984 (*n* > 3; ^***^p < 0.001).

Prevention of biofilm growth was examined in MRSA Mu50 (Figures 3A,B) and *S. epidermidis* ATCC 35984 (Figures 3C,D) with the xCELLigence RTCA system over 48 h. A high cell index (CI) correlates with bacteria attaching to the gold electrodes located at the bottom of the well (Abrantes and Africa, 2020). For both *S. aureus* and *S. epidermidis*, the untreated control showed a high increase in CI within the first 12 h, reaching a CI of 0.32 in MRSA Mu50 (Figure 3A) and 0.25 in *S. epidermidis* ATCC 35984 (Figure 3C), before steadily increasing at a slower rate to reach 0.5 in MRSA Mu50 and 0.45 in *S. epidermidis* ATCC 35984 after 48 h. Monotherapy of DDC (8 μg/ml) and Cu^2+^ (32 μg/ml) resulted in a faster CI increase compared to the untreated control, reaching a maximum after 5 h in MRSA Mu50 (CI: DDC = 0.40; Cu^2+^ = 0.19) and 6 h in *S. epidermidis* ATCC 35984 (CI: DDC = 0.35; Cu^2+^ = 0.18). The fast CI increase of DDC or Cu^2+^ treated bacteria should not be a result of DDC or Cu^2+^ salts interacting with the gold electrodes or the impedance, as these were assessed with the baselines. The initial increased bacterial attachment when treated with DDC or Cu^2+^ can be explained by the subinhibitory concentration of DDC or Cu^2+^ alone used in this experiment. Treatment with DDC and Cu^2+^ can induce oxidative stress and the production of reactive oxygen species in *S. aureus*, which play a role in the control of different cellular processes, such as biofilm formation (Seixas et al., 2021). Treatment with DDC alone showed no significant difference from the mean CI (12–48 h) compared to the untreated control (CI: 0.44 MRSA Mu50 and 0.38 *S. epidermidis* ATCC 35984). Treatment with Cu^2+^ alone resulted in approximately half the CI compared to untreated control (CI: 0.22 MRSA Mu50 and 0.18 *S. epidermidis* ATCC 35984), translating in less bacteria attaching to the bottom of the well and forming biofilms. Lastly, treatment with DDC-Cu^2+^ (8 μg/ml DDC + 32 μg/ml Cu^2+^) resulted in a CI of 0 after 12 h and a mean CI of 0.04 and 0.03 after 48 h in MRSA Mu50 (Figure 3B) and *S. epidermidis* ATCC 35984 (Figure 3D), respectively. Therefore, treatment with DDC-Cu^2+^ prevented the attachment of bacteria over 48 h, which can be a result of high bacterial killing at the tested concentrations. To determine if bacterial killing was responsible for prevention of bacterial attachment, lower DDC-Cu^2+^ concentrations can be investigated.

### DDC-Cu^2+^ inhibits biofilm growth

**Figure 4:**
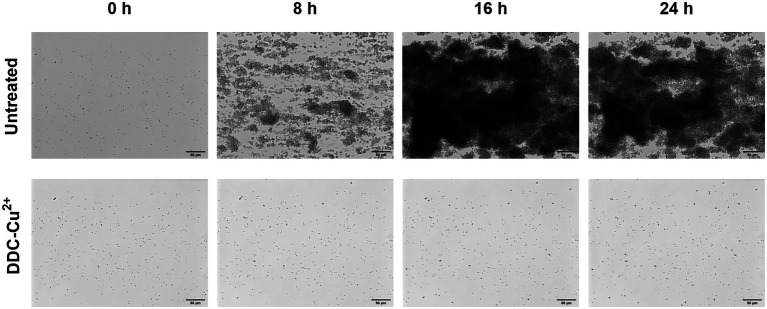
Monitoring of MRSA Mu50 biofilm formation over 24 h when left untreated or treated with a combination of 8 μg/ml diethyldithiocarbamate and 32 μg/ml Cu^2+^ combination (DDC-Cu^2+^) using the Bioflux system. Scale bar represents 50 μm.

Similar results were observed with the Bioflux system (Figure 4). In the untreated control, under constant nutrient flow, MRSA Mu50 bacteria started to aggregate within 8 h, formed biofilms within 16 h that continuously increased in size within 24 h (Figure 4, top time lapse). When DDC-Cu^2+^ was added to the constant nutrient flow, inhibition of biofilm growth was achieved over 24 h (Figure 4; bottom time lapse. Supplementary File 1: Video footage). Similar observations were made in *S. epidermidis* ATCC 35984 biofilms (data not shown).

### Cytotoxicity of DDC-Cu^2+^ *in vitro*

**Figure 5:**
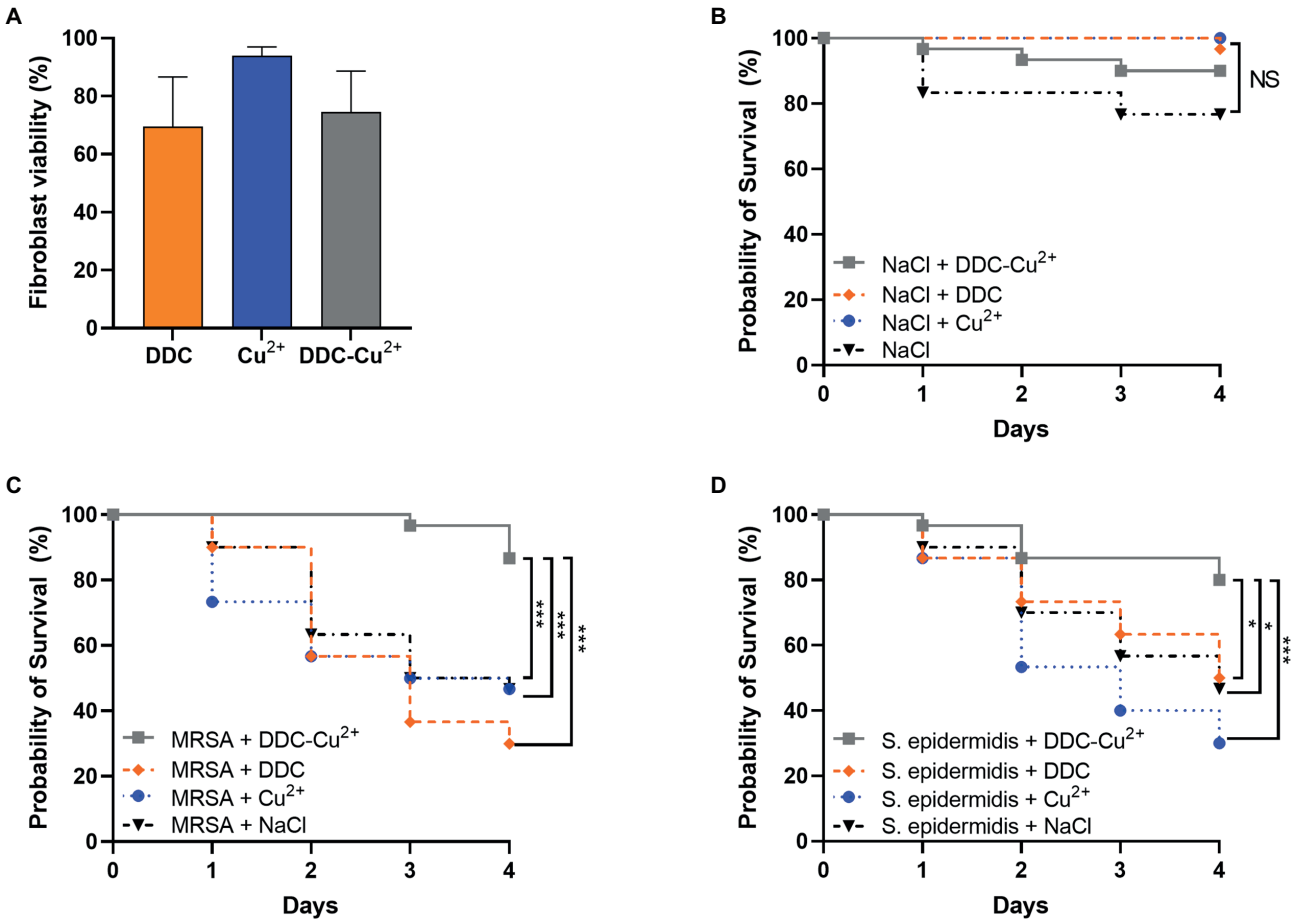
Effect of diethyldithiocarbamate [DDC; orange; 8 μg/ml **(A)**, 6.4 mg/kg **(B–D)**], Cu^2+^ [blue; 32 μg/ml **(A)**, 25.6 mg/kg **(B–D)**] and DDC-Cu^2+^ (grey) on **(A)** fibroblast viability (*n* = 3), on **(B)** probability of *Galleria mellonella* survival (30/group; *n* = 120), on the probability of survival of *Galleria mellonella* infected with **(C)** MRSA Mu50 (30/group; *n* = 120), and **(D)** infected with *S. epidermidis* ATCC 35984 (30/group; *n* = 120; NS = not significant; ^*^p < 0.05; ^***^p < 0.001).

The *in vitro* cytotoxicity of the compounds was investigated in fibroblast cells over 18 h (Figure 5A). Monotherapy with DDC and Cu^2+^ showed 70 and 94% fibroblast viability, respectively. Treatment with DDC-Cu^2+^ resulted in 75% fibroblast viability, showing no difference compared to DDC monotherapy.

### Toxicity and efficacy of DDC-Cu^2+^ *in vivo* using *Galleria mellonella* larvae

To investigate potential toxic treatment effects *in vivo*, *G. mellonella* larvae were injected with DDC, Cu^2+^, DDC-Cu^2+^ or vehicle control (saline) and the survival was monitored over 4 days. DDC, Cu^2+^ and DDC-Cu^2+^ showed similar survival rates as the vehicle control, indicating no treatment toxicity in *G. mellonella* (Figure 5B).

To assess the antimicrobial activity of DDC-Cu^2+^ *in vivo*, the survival of MRSA- or *S. epidermidis-*infected *G. mellonella* was examined over 4 days. In infected larvae, treatment with DDC or Cu^2+^ resulted in a poor survival rate, similar to the vehicle control for both MRSA- and *S. epidermidis*-infected *G. mellonella* (*p* > 0.05; Figures 5C,D, respectively). However, MRSA-infected and DDC-Cu^2+^ treated larvae, displayed a significantly higher survival rate of 87% (26/30 larvae) compared to MRSA-infected, vehicle control larvae that showed 47% survival (14/30 larvae, *p* = 0.0004; Figure 5C). Moreover, the survival rate of MRSA-infected, DDC-Cu^2+^ treated larvae was significantly higher compared to treatment with DDC alone (9/30 larvae; *p* = 0.0003) or Cu^2+^ alone (14/30 larvae; *p* = 0.0003). Similar results were found in *S. epidermidis*-infected *G. mellonella*, which showed a significantly higher survival rate of 80% (24/30 larvae) for *S. epidermidis*-infected, DDC-Cu^2+^ treated larvae compared to 47% survival (14/30 larvae) for *S. epidermidis*-infected, vehicle control larvae (*p* = 0.0152; Figure 5D). Survival of *S. epidermidis*-infected, DDC-Cu^2+^ treated *G. mellonella* (26/30 larvae) was also significantly higher compared to mono treatment with DDC (15/30 larvae; *p* = 0.0152) or Cu^2+^ (9/30 larvae; *p* = 0.0003).

## Discussion

DDC is the metabolite of disulfiram, an FDA-approved drug for the oral treatment of chronic alcoholism, that has been previously investigated for its activity against fungi (Harrison et al., 2007; De Brucker et al., 2013), parasites (Khouri et al., 2010; Celes et al., 2016) and bacteria (Taylor et al., 1987; Dalecki et al., 2015; Long, 2017; Sheppard et al., 2018; Frazier et al., 2019). In the current study, DDC was repurposed and combined with Cu^2+^ for pre-clinical validation as a novel antibacterial treatment. Confirming previous results, DDC showed limited antibacterial activity against *S. aureus* and *S. epidermidis*, with MICs ranging from 16 to above 32 μg/ml and no growth inhibition of Gram-negative bacteria with MICs above 64 μg/ml. The lack of antibacterial activity of DDC against *E. coli* and *P. aeruginosa* was explained by the elevated presence of glutathione in Gram-negative bacteria. Cellular glutathione interacts with DDC and disulfiram by thiol-disulfide exchange reaction (Long, 2017; Frazier et al., 2019). While monotherapy with disulfiram showed antibacterial and antibiofilm activity against *S. aureus in vitro* and *in vivo* and synergized with multiple antibiotics (Long, 2017; Thakare et al., 2019), these results were not observed with the *in vivo* formed metabolites of disulfiram (Frazier et al., 2019). As disulfiram is hypothesized to form disulfides with thiophilic residues of bacterial cofactors, metabolites and enzymes (Long, 2017; Sheppard et al., 2018), the lack of antibacterial activity of DDC and other metabolites can be explained by lack of thiol-disulfide exchange. In addition, disulfiram and DDC differentiate in their chemical and physical properties (Gessner and Gessner, 1992). While disulfiram shows poor water solubility and physiological instability, therefore limiting local clinical applications (Xie et al., 2022), DDC is highly water soluble (Gessner and Gessner, 1992), a labile molecule and a very strong metal chelator (Butcher et al., 2018). Specifically, Cu^2+^ was investigated, as disulfiram dissociates in the presence of Cu^2+^, to form DDC, which chelates the metal ion and forms the stable Cu(DDC)_2_ complex that can be visualized by a color change (Dalecki et al., 2015) and has been shown to result in anticancer activity (Viola-Rhenals et al., 2018).

Dalecki et al. (2015) were the first to reveal that disulfiram and DDC displayed antimycobacterial effects only in the presence of Cu^2+^, as the presence of iron and zinc ions did not increase the antimicrobial activity of DDC against *Mycobacterium tuberculosis*. In addition, 90% of *Mycobacterium tuberculosis* inhibition occurred with 0.3 μM disulfiram, equivalent to 0.6 μM DDC and 0.3 μM Cu^2+^, which is consistent with the molar ratio of 2:1 and consequently the formation of the Cu(DDC)_2_ complex (Dalecki et al., 2015). Based on these results, Saputo et al. (2018) investigated the effect of disulfiram with Cu^2+^ on *Streptococcus mutans* and observed a reduction of disulfiram MIC from 16 μg/ml to 4 μg/ml (equivalent to 8 μg/ml DDC) in the presence of 106.6 μg/ml Cu^2+^. The concentration of disulfiram required to inhibit *S. mutans* biofilm formation was even lower with 2 μg/ml (equivalent to 4 μg/ml DDC) in the presence of 106.6 μg/ml Cu^2+^, resulting in synergistic effects of disulfiram and Cu^2+^ against both the planktonic and biofilm forms. We obtained comparable results against *S. aureus* and *S. epidermidis*, with concentrations as low as 0.5 μg/ml DDC and 2 μg/ml Cu^2+^ against planktonic MRSA Mu50 and 4 μg/ml DDC and 16 μg/ml Cu^2+^ against biofilm MRSA Mu50, respectively, reaching synergistic effects in both forms. In contrast to the concentrations required for the antimycobacterial activity, the Cu^2+^ concentrations necessary to enhance the activity of DDC against *S. mutans*, *S. aureus* and *S. epidermidis* exceeded the DDC concentration. This concentration-dependent antibacterial activity was also observed by Menghani et al. (2021) against *Streptococcus pneumoniae*.

The concentrations of DDC and Cu^2+^ play an important role in the proposed mode of action for the antibacterial activity of DDC-Cu^2+^. The mechanism of DDC can in part be explained by inhibition of the *S. aureus* carbonic anhydrase (Urbanski et al., 2021) and the chelation and extraction of required metal cofactors, including Cu^2+^ from metallo-enzymes, such as superoxide dismutase, rendering bacteria more susceptible to oxidative stress (Frazier et al., 2019). In addition, at high levels Cu^2+^ is toxic by the generation of reactive oxygen species through the Cu^+^/Cu^2+^ redox cycle and by competing with other metals at the enzymatic binding sites, leading to the inactivation and oxidation of free thiol groups of various proteins (Baker et al., 2010; Dupont et al., 2011). Therefore, bacteria have developed mechanisms to regulate the intracellular copper concentration and to evade copper induced toxicity, staphylococci have efflux systems in form of a P1-type ATPase transporter, copper-binding chaperones and copper-responsive regulators (Solioz, 2018), explaining the low antimicrobial activity of Cu^2+^ with a MIC above 128 μg/ml.

**Figure 6:**
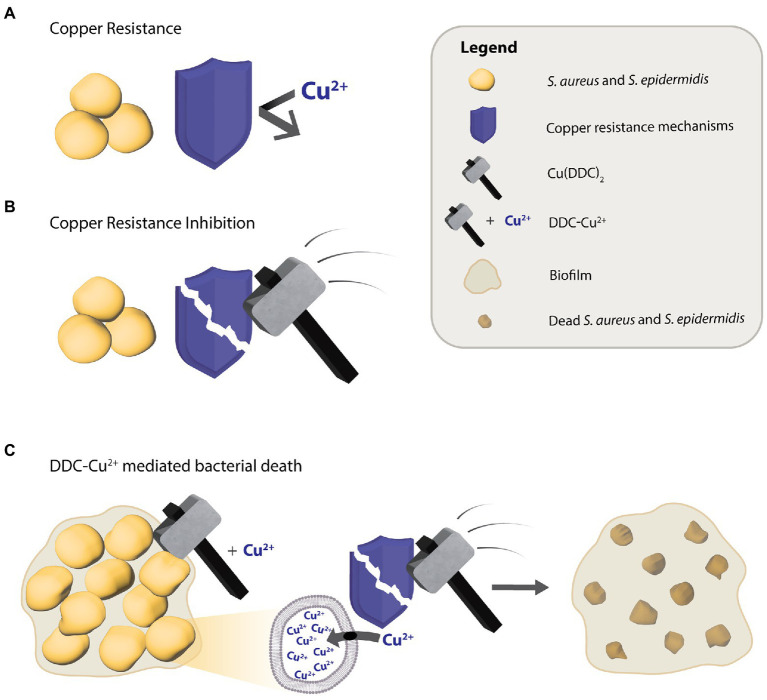
Putative diethyldithiocarbamate and copper (DDC-Cu^2+^) mode of action against *S. aureus* and *S. epidermidis*. **(A)** The antibacterial activity of Cu^2+^ is limited by copper resistance mechanisms of bacteria. **(B)** The Cu(DDC)_2_ complex inhibits the bacterial copper resistance mechanism but does not kill bacteria. **(C)** The combination of Cu(DDC)_2_ complex and excessive Cu^2+^ (called DDC-Cu^2+^) effectively kills bacteria, as the Cu(DDC)_2_ complex inhibits the copper resistance mechanisms, allowing for the excess Cu^2+^ to increase copper induced toxicity.

To explain the mode of action behind the antimycobacterial activity of DDC and Cu^2+^, Dalecki et al. (2015) proposed a Trojan Horse model, where the Cu(DDC)_2_ complex transports Cu^2+^ into the cytoplasm, thereby protecting Cu^2+^ from the bacterial copper resistance mechanisms, which in turn, allows access to targets that usually are not available to free Cu^2+^ (Dalecki et al., 2015). However, in the present study the concentrations closest to corresponding to the Cu(DDC)_2_ complex, 8 μg/ml DDC with 4 μg/ml Cu^2+^ and 32 μg/ml DDC with 16 μg/ml Cu^2+^, resulted in less than 25% *S. aureus* and *S. epidermidis* biofilm killing. Therefore, the antibiofilm activity of DDC and Cu^2+^ against *S. aureus* and *S. epidermidis* could not exclusively be associated to the Cu(DDC)_2_-complex. The lowest concentration of the mix leading to a statistical increase in *S. aureus* and *S. epidermidis* biofilm killing compared to monotherapy with Cu^2+^ was 8 μg/ml DDC in combination with 32 μg/ml Cu^2+^. Hence, the antibacterial activity of DDC-Cu^2+^ against *S. aureus* and *S. epidermidis* seems to be based on the formation of the Cu(DDC)_2_ complex and an excess of free Cu^2+^. Based on these results, we hypothesize that the Cu(DDC)_2_ complex inhibits at least one of the copper homeostasis components such as the efflux transporter, allowing for the additional Cu^2+^ to accumulate within the bacteria and cause copper induced toxicity (Figure 6). In addition, the extensive inhibition of MRSA biofilm attachment and aggregation by DDC-Cu^2+^ observed with the xCELLigence and the Bioflux systems depended on the combination of DDC and Cu^2+^ and can be caused by excess Cu^2+^ that represses the expression of positive biofilm formation regulators, such as *agr* and *sae* (Baker et al., 2010).

While the DDC-Cu^2+^ combination of 8 μg/ml DDC and 32 μg/ml Cu^2+^ inhibited planktonic *S. aureus* and *S. epidermidis* growth and biofilm formation, the same concentrations showed low cytotoxic effects against fibroblasts. As antimicrobial and cytotoxic results obtained *in vitro* do not always accurately predict activity under *in vivo* conditions (Tsai et al., 2016), both the antibacterial activity and the toxicity of DDC-Cu^2+^ was investigated using the *G. mellonella* model. These larvae have been shown to be good models to assess the safety and efficacy of antimicrobial agents against *S. aureus* (Brackman et al., 2011; Desbois and Coote, 2011; Tsai et al., 2016). The high survival rate of uninfected, treated larvae confirmed the non-toxicity of DDC-Cu^2+^ and the significant increase of survival of MRSA- and *S. epidermidis-*infected, DDC-Cu^2+^ treated *G. mellonella* confirmed the *in vitro* antibacterial activity. To the best of our knowledge, this is the first study to report the antibacterial activity and non-toxicity of DDC in combination with Cu^2+^ in the *G. mellonella* model. The promising results obtained with the *G. mellonella* model pre-screening experiment increases the confidence in the performance of Cu(DDC)_2_ and excess Cu^2+^ to progress to preclinical mammalian models. A pharmaceutical development of the DDC-Cu^2+^ combination is ongoing to provide a drug delivery platform for the treatment of infected wounds and for surgical applications. A DDC-Cu^2+^ formulation has potential to synergistically enhance standard-of-care with oral or topical antibiotics and reduce the pressure on resistance development.

In conclusion, the combination of DDC-Cu^2+^ showed considerable *in vitro* antimicrobial activity against planktonic and biofilm cultures of *S. aureus* and *S. epidermidis*. By enhancing multiple antibiotic classes, preventing biofilm formation, showing non-toxicity and antibacterial activity *in vivo*, the DDC-Cu^2+^ combination represents an effective novel treatment strategy to control *S. aureus* and *S. epidermidis* biofilms. Ongoing studies are focused on developing drug delivery platforms containing the DDC-Cu^2+^ combination for clinical application and to determine whether similar safety and antimicrobial efficacy can be observed in other *in vivo* models of infection.

## Data availability statement

The raw data supporting the conclusions of this article will be made available by the authors, without undue reservation.

## Author contributions

LK designed and conducted the experiments, analyzed data, and wrote the manuscript. KR designed and conducted the experiments and analyzed data. AA analyzed data, contributed to the result section, and reviewed the manuscript. TC, BK, and MH provided technical assistance for some of the experiments and reviewed the manuscript. AZ, RS, and KR supervised the study, reviewed the manuscript and are ranked in ascending order of contribution with KR as senior author. All authors contributed to the article and approved the submitted version.

## Funding

This work was supported by the National Health and Medical Research Council (GNT1163634 and GNT2004036), the University of Adelaide (Joint PhD Scholarship held by LK) and The Hospital Research Foundation, Australia. Bioflux Z1000 setup was (partially) funded by Netherlands Scientific Organisation (NWO) Earth and Life Sciences (ALW), grant 834.13.006 to BK.

## Conflict of interest

KR holds intellectual property on the DDC-Cu^2+^ treatment (PCT/AU2020/050661).

The remaining authors declare that the research was conducted in the absence of any commercial or financial relationships that could be construed as a potential conflict of interest.

## Publisher’s note

All claims expressed in this article are solely those of the authors and do not necessarily represent those of their affiliated organizations, or those of the publisher, the editors and the reviewers. Any product that may be evaluated in this article, or claim that may be made by its manufacturer, is not guaranteed or endorsed by the publisher.

## Supplementary material

Supplementary Figure 1 Confocal microscopy images of stained MRSA Mu50 biofilms with LIVE/DEAD BacLight staining after treatment with 8 μg/ml diethyldithiocarbamate and 32 μg/ml Cu^2+^. Confocal microscopy images results: green = viable bacteria; red = dead bacteria. **(A)** Untreated MRSA Mu50 biofilm at 20 ×. **(B)** MRSA Mu50 biofilm after treatment with DDC-Cu^2+^ at 20 ×.

Supplementary File 1 Video footage of MRSA Mu50 biofilm formation over 24 h when exposed to constant nutrient flow or when nutrient flow is supplemented with a combination of diethyldithiocarbamate and Cu^2+^ combination (DDC-Cu^2+^) using the Bioflux system. Scale bar represents 50 μm, 8 FPS corresponding to 2 h/s.

## References

[ref1] Abrantes P. Africa C. W. J. (2020). Measuring *Streptococcus mutans*, *Streptococcus sanguinis* and *Candida albicans* biofilm formation using a real-time impedance-based system. J. Microbiol. Methods 169:105815. doi: 10.1016/j.mimet.2019.105815, PMID: 31870585

[ref2] Anderson D. J. Sexton D. J. Kanafani Z. A. Auten G. Kaye K. S. (2007). Severe surgical site infection in community hospitals: epidemiology, key procedures, and the changing prevalence of methicillin-resistant *Staphylococcus aureus*. Infect. Control Hosp. Epidemiol. 28, 1047–1053. doi: 10.1086/520731, PMID: 17932825

[ref3] Australian Commission on Safety and Quality in Health Care (ACSQHC). (2019). AURA 2019: Third Australian Report on Antimicrobial Use and Resistance in Human Health. Sydney: ACSQHC.

[ref4] Baker J. Sitthisak S. Sengupta M. Johnson M. Jayaswal R. K. Morrissey J. A. (2010). Copper stress induces a global stress response in *Staphylococcus aureus* and represses *sae* and *agr* expression and biofilm formation. Appl. Environ. Microbiol. 76, 150–160. doi: 10.1128/AEM.02268-09, PMID: 19880638 PMC2798663

[ref5] Brackman G. Cos P. Maes L. Nelis H. J. Coenye T. (2011). Quorum sensing inhibitors increase the susceptibility of bacterial biofilms to antibiotics in vitro and in vivo. Antimicrob. Agents Chemother. 55, 2655–2661. doi: 10.1128/AAC.00045-11, PMID: 21422204 PMC3101409

[ref6] Butcher K. Kannappan V. Kilari R. S. Morris M. R. McConville C. Armesilla A. L.. (2018). Investigation of the key chemical structures involved in the anticancer activity of disulfiram in A549 non-small cell lung cancer cell line. BMC Cancer 18:753. doi: 10.1186/s12885-018-4617-x, PMID: 30031402 PMC6054747

[ref7] Cahill T. J. Baddour L. M. Habib G. Hoen B. Salaun E. Pettersson G. B.. (2017). Challenges in infective endocarditis. J. Am. Coll. Cardiol. 69, 325–344. doi: 10.1016/j.jacc.2016.10.066 28104075

[ref8] Celes F. S. Trovatti E. Khouri R. Van Weyenbergh J. Ribeiro S. J. Borges V. M.. (2016). DETC-based bacterial cellulose bio-curatives for topical treatment of cutaneous leishmaniasis. Sci. Rep. 6:38330. doi: 10.1038/srep38330, PMID: 27922065 PMC5138610

[ref9] Costerton J. W. Stewart P. S. Greenberg E. P. (1999). Bacterial biofilms: a common cause of persistent infections. Science 284, 1318–1322. doi: 10.1126/science.284.5418.1318 10334980

[ref10] Crabbé A. Jensen P. Ø. Bjarnsholt T. Coenye T. (2019). Antimicrobial tolerance and metabolic adaptations in microbial biofilms. Trends Microbiol. 27, 850–863. doi: 10.1016/j.tim.2019.05.003, PMID: 31178124

[ref11] Craft K. M. Nguyen J. M. Berg L. J. Townsend S. D. (2019). Methicillin-resistant *Staphylococcus aureus* (MRSA): antibiotic-resistance and the biofilm phenotype. Medchemcomm. 10, 1231–1241. doi: 10.1039/C9MD00044E, PMID: 31534648 PMC6748282

[ref12] Dalecki A. G. Haeili M. Shah S. Speer A. Niederweis M. Kutsch O.. (2015). Disulfiram and copper ions kill mycobacterium tuberculosis in a synergistic manner. Antimicrob. Agents Chemother. 59, 4835–4844. doi: 10.1128/AAC.00692-15, PMID: 26033731 PMC4505271

[ref13] De Brucker K. Bink A. Meert E. Cammue B. P. Thevissen K. (2013). Potentiation of antibiofilm activity of amphotericin B by superoxide dismutase inhibition. Oxidative Med. Cell. Longev. 2013:704654, 1–7. doi: 10.1155/2013/704654 PMC3774027 24078861

[ref14] Desbois A. P. Coote P. J. (2011). Wax moth larva (*Galleria mellonella*): an in vivo model for assessing the efficacy of antistaphylococcal agents. J. Antimicrob. Chemother. 66, 1785–1790. doi: 10.1093/jac/dkr198, PMID: 21622972

[ref15] Dinarvand M. Spain M. P. Vafaee F. (2020). Pharmacodynamic functions of synthetic derivatives for treatment of methicillin-resistant *Staphylococcus aureus* (MRSA) and mycobacterium tuberculosis. Front. Microbiol. 11:551189. doi: 10.3389/fmicb.2020.551189, PMID: 33329419 PMC7729195

[ref16] Dupont C. L. Grass G. Rensing C. (2011). Copper toxicity and the origin of bacterial resistance - new insights and applications. Metallomics 3, 1109–1118. doi: 10.1039/c1mt00107h, PMID: 21984219

[ref17] European Centre for Disease Prevention and Control (2022). Antimicrobial Resistance in the EU/EEA (EARS-Net) – Annual Epidemiological Report 2020. Stockholm: ECDC.

[ref18] Feng T. Leptihn S. Dong K. Loh B. Zhang Y. Stefan M. I.. (2021). JD419, a *Staphylococcus aureus* phage with a unique morphology and broad host range. Front. Microbiol. 12:602902. doi: 10.3389/fmicb.2021.602902, PMID: 33967969 PMC8100676

[ref19] Frazier K. R. Moore J. A. Long T. E. (2019). Antibacterial activity of disulfiram and its metabolites. J. Appl. Microbiol. 126, 79–86. doi: 10.1111/jam.14094, PMID: 30160334

[ref20] Gessner P. K. Gessner T. (1992). Toxicology. Disulfiram and its metabolite diethyldithiocarbamate pharmacology and status in the treatment of alcoholism, HIV infections, AIDS and heavy metal toxicity. London, United Kingdom: Chapman and Hall, 335–345

[ref21] Gessner P. K. Gessner T. (1992). “Relevant physical and chemical properties” in Disulfiram and its metabolite, diethyldithiocarbamate: pharmacology and status in the treatment of alcoholism, HIV infections, AIDS and heavy metal toxicity. eds. Gessner P. K. Gessner T. (Springer Netherlands: Dordrecht), 7–12.

[ref22] Harrison J. J. Turner R. J. Ceri H. (2007). A subpopulation of *Candida albicans* and Candida tropicalis biofilm cells are highly tolerant to chelating agents. FEMS Microbiol. Lett. 272, 172–181. doi: 10.1111/j.1574-6968.2007.00745.x, PMID: 17490429

[ref23] Hoogenkamp M.A. (2021). Challenging dental unit water biofilms. Amsterdam, The Netherlands: University of Amsterdam.

[ref24] Kaul L. Süss R. Zannettino A. Richter K. (2021). The revival of dithiocarbamates: from pesticides to innovative medical treatments. iScience 24:102092. doi: 10.1016/j.isci.2021.102092, PMID: 33598645 PMC7868997

[ref25] Kaushik A. Kest H. (2018). Pediatric methicillin-resistant *Staphylococcus aureus* osteoarticular infections. Microorganisms 6:40. doi: 10.3390/microorganisms6020040, PMID: 29734665 PMC6027280

[ref26] Khouri R. Novais F. Santana G. de Oliveira C. I. Vannier dos Santos M. A. Barral A.. (2010). DETC induces leishmania parasite killing in human *in vitro* and murine *in vivo* models: a promising therapeutic alternative in leishmaniasis. PLoS One 5:e14394. doi: 10.1371/journal.pone.0014394, PMID: 21200432 PMC3006171

[ref27] Kleinschmidt S. Huygens F. Faoagali J. Rathnayake I. U. Hafner L. M. (2015). *Staphylococcus* epidermidis as a cause of bacteremia. Future Microbiol. 10, 1859–1879. doi: 10.2217/fmb.15.98 26517189

[ref28] Lee J. Y. H. Monk I. R. Gonçalves da Silva A. Seemann T. Chua K. Y. L. Kearns A.. (2018). Global spread of three multidrug-resistant lineages of *Staphylococcus epidermidis*. Nat. Microbiol. 3, 1175–1185. doi: 10.1038/s41564-018-0230-7 30177740 PMC6660648

[ref29] Liu C. Bayer A. Cosgrove S. E. Daum R. S. Fridkin S. K. Gorwitz R. J.. (2011). Clinical practice guidelines by the Infectious Diseases Society of America for the treatment of methicillin-resistant *Staphylococcus aureus* infections in adults and children. Clin. Infect. Dis. 52, e18–e55. doi: 10.1093/cid/ciq146, PMID: 21208910

[ref30] Long T. E. (2017). Repurposing thiram and disulfiram as antibacterial agents for multidrug-resistant *Staphylococcus aureus* infections. Antimicrob. Agents Chemother. 61, e00898-17. doi: 10.1128/AAC.00898-17 28674046 PMC5571293

[ref31] López-Cortés L. E. Gálvez-Acebal J. Rodríguez-Baño J. (2020). Therapy of *Staphylococcus aureus* bacteremia: evidences and challenges. Enferm. Infecc. Microbiol. Clin. (Engl. Ed). 38, 489–497. doi: 10.1016/j.eimc.2020.01.018, PMID: 32169398

[ref32] Mah T. F. O'Toole G. A. (2001). Mechanisms of biofilm resistance to antimicrobial agents. Trends Microbiol. 9, 34–39. doi: 10.1016/S0966-842X(00)01913-2 11166241

[ref33] Menghani S. V. Rivera A. Neubert M. Hagerty J. R. Lewis L. Galgiani J. N.. (2021). Demonstration of N,N-dimethyldithiocarbamate as a copper-dependent antibiotic against multiple upper respiratory tract pathogens. Microbiol. Spectr. 9:e0077821. doi: 10.1128/Spectrum.00778-21, PMID: 34468162 PMC8557878

[ref34] Nishimori I. Vullo D. Minakuchi T. Scozzafava A. Osman S. M. AlOthman Z.. (2014). Anion inhibition studies of two new β-carbonic anhydrases from the bacterial pathogen legionella pneumophila. Bioorg. Med. Chem. Lett. 24, 1127–1132. doi: 10.1016/j.bmcl.2013.12.124, PMID: 24461298

[ref35] Otto M. (2008). Staphylococcal biofilms. Curr. Top. Microbiol. Immunol. 322, 207–228. doi: 10.1007/978-3-540-75418-3_10, PMID: 18453278 PMC2777538

[ref36] Patiniott P. Jacombs A. Kaul L. Hu H. Warner M. Klosterhalfen B.. (2022). Are late hernia mesh complications linked to staphylococci biofilms? Hernia. doi: 10.1007/s10029-022-02583-0 [Epub ahead of print]., PMID: 35286510 PMC9525333

[ref37] Peeters E. Nelis H. J. Coenye T. (2008). Comparison of multiple methods for quantification of microbial biofilms grown in microtiter plates. J. Microbiol. Methods 72, 157–165. doi: 10.1016/j.mimet.2007.11.010, PMID: 18155789

[ref38] Phillips M. G. M. Nedunchezian D. Lukrec A. Howard R. G. (1991). Disulfiram inhibits the in vitro growth of methicillin-resistant *Staphylococcus aureus*. Antimicrob. Agents Chemother. 35, 785–787. doi: 10.1128/AAC.35.4.785, PMID: 2069390 PMC245102

[ref39] Pushpakom S. Iorio F. Eyers P. A. Escott K. J. Hopper S. Wells A.. (2018). Drug repurposing: progress, challenges and recommendations. Nat. Rev. Drug Discov. 18, 41–58. doi: 10.1038/nrd.2018.168 30310233

[ref40] Richter K. (2019). Tackling superbugs in their slime castles: innovative approaches against antimicrobial-resistant biofilm infections. Microbiol. Aust. 40, 165–168. doi: 10.1071/MA19049

[ref41] Richter K. Facal P. Thomas N. Vandecandelaere I. Ramezanpour M. Cooksley C.. (2017a). Taking the silver bullet colloidal silver particles for the topical treatment of biofilm-related infections. ACS Appl. Mater. Interfaces 9, 21631–21638. doi: 10.1021/acsami.7b03672, PMID: 28598149

[ref42] Richter K. Ramezanpour M. Thomas N. Prestidge C. A. Wormald P. J. Vreugde S. (2016). Mind “De GaPP”: *in vitro* efficacy of deferiprone and gallium-protoporphyrin against *Staphylococcus aureus* biofilms. Int. Forum. Allergy Rhinol. 6, 737–743. doi: 10.1002/alr.21735, PMID: 26919404

[ref43] Richter K. Thomas N. Claeys J. McGuane J. Prestidge C. A. Coenye T.. (2017b). A topical hydrogel with deferiprone and gallium-protoporphyrin targets bacterial iron metabolism and has antibiofilm activity. Antimicrob. Agents Chemother. 61, e00481-17. doi: 10.1128/AAC.00481-17 28396543 PMC5444117

[ref44] Sánchez-López E. Gomes D. Esteruelas G. Bonilla L. Lopez-Machado A. L. Galindo R.. (2020). Metal-based nanoparticles as antimicrobial agents: an overview. Nano 10:292. doi: 10.3390/nano10020292, PMID: 32050443 PMC7075170

[ref45] Santajit S. Indrawattana N. (2016). Mechanisms of antimicrobial resistance in ESKAPE pathogens. Biomed. Res. Int. 2016, 1–8. doi: 10.1155/2016/2475067 PMC4871955 27274985

[ref46] Saputo S. Faustoferri R. C. Quivey R. G. Jr. (2018). A drug repositioning approach reveals that *Streptococcus mutans* is susceptible to a diverse range of established antimicrobials and nonantibiotics. Antimicrob. Agents Chemother. 62, e01674-17. doi: 10.1128/AAC.01674-17 29061736 PMC5740335

[ref47] Seixas A. F. Quendera A. P. Sousa J. P. Silva A. F. Q. Arraiano C. M. Andrade J. M. (2021). Bacterial response to oxidative stress and RNA oxidation. Front. Genet. 12:821535. doi: 10.3389/fgene.2021.821535 35082839 PMC8784731

[ref48] Sheppard J. G. Frazier K. R. Saralkar P. Hossain M. F. Geldenhuys W. J. Long T. E. (2018). Disulfiram-based disulfides as narrow-spectrum antibacterial agents. Bioorg. Med. Chem. Lett. 28, 1298–1302. doi: 10.1016/j.bmcl.2018.03.023, PMID: 29571571 PMC5893419

[ref49] Solioz M. (2018). Copper homeostasis in gram-positive bacteria. Copper and bacteria. London: Springer Briefs in Biometals, 21–48.

[ref50] Sopirala M. M. Mangino J. E. Gebreyes W. A. Biller B. Bannerman T. Balada-Llasat J. M.. (2010). Synergy testing by Etest, microdilution checkerboard, and time-kill methods for pan-drug-resistant *Acinetobacter baumannii*. Antimicrob. Agents Chemother. 54, 4678–4683. doi: 10.1128/AAC.00497-10, PMID: 20713678 PMC2976112

[ref51] Sovari S. N. Vojnovic S. Bogojevic S. S. Crochet A. Pavic A. Nikodinovic-Runic J.. (2020). Design, synthesis and in vivo evaluation of 3-arylcoumarin derivatives of rhenium(I) tricarbonyl complexes as potent antibacterial agents against methicillin-resistant *Staphylococcus aureus* (MRSA). Eur. J. Med. Chem. 205:112533. doi: 10.1016/j.ejmech.2020.112533, PMID: 32739550

[ref52] Stevens D. L. Bisno A. L. Chambers H. F. Everett E. D. Dellinger P. Goldstein E. J.. (2005). Practice guidelines for the diagnosis and management of skin and soft-tissue infections. Clin. Infect. Dis. 41, 1373–1406. doi: 10.1086/497143 16231249

[ref53] Taylor E. H. Walker E. Bartelt M. Day S. Pappas A. (1987). *In vitro* antimicrobial activity of diethyldithiocarbamate and dimethyldithiocarbamate against methicillin-resistant *Staphylococcus*. Ann. Clin. Lab. Sci. 17, 171–177. PMID: 3037985

[ref54] Thakare R. Shukla M. Kaul G. Dasgupta A. Chopra S. (2019). Repurposing disulfiram for treatment of *Staphylococcus aureus* infections. Int. J. Antimicrob. Agents 53, 709–715. doi: 10.1016/j.ijantimicag.2019.03.024, PMID: 30954635

[ref55] Thangamani S. Mohammad H. Younis W. Seleem M. N. (2015). Drug repurposing for the treatment of staphylococcal infections. Curr. Pharm. Des. 21, 2089–2100. doi: 10.2174/1381612821666150310104416, PMID: 25760334 PMC8672279

[ref56] Tong S. Y. C. Davis J. S. Eichenberger E. Holland T. L. Fowler V. G. Jr. (2015). *Staphylococcus aureus* infections: epidemiology, pathophysiology, clinical manifestations, and management. Clin. Microbiol. Rev. 28, 603–661. doi: 10.1128/CMR.00134-14, PMID: 26016486 PMC4451395

[ref57] Tsai C. J.-Y. Loh J. M. S. Proft T. (2016). *Galleria mellonella* infection models for the study of bacterial diseases and for antimicrobial drug testing. Virulence 7, 214–229. doi: 10.1080/21505594.2015.1135289, PMID: 26730990 PMC4871635

[ref58] Urbanski L. J. Vullo D. Parkkila S. Supuran C. T. (2021). An anion and small molecule inhibition study of the β-carbonic anhydrase from *Staphylococcus aureus*. J. Enzyme Inhib. Med. Chem. 36, 1088–1092. doi: 10.1080/14756366.2021.1931863, PMID: 34056990 PMC8168783

[ref59] Viola-Rhenals M. Patel K. R. Jaimes-Santamaria L. Wu G. Liu J. Dou Q. P. (2018). Recent advances in antabuse (disulfiram): the importance of its metal-binding ability to its anticancer activity. Curr. Med. Chem. 25, 506–524. doi: 10.2174/0929867324666171023161121, PMID: 29065820 PMC6873226

[ref60] Walsh L. Johnson C. N. Hill C. Ross R. P. (2021). Efficacy of phage- and bacteriocin-based therapies in combatting nosocomial MRSA infections. Front. Mol. Biosci. 8:654038. doi: 10.3389/fmolb.2021.654038, PMID: 33996906 PMC8116899

[ref61] Wang X. Bittner T. Milanov M. Kaul L. Mundinger S. Koch H.-G.. (2021). Pyridinium modified anthracenes and their endoperoxides provide a tunable scaffold with activity against gram-positive and gram-negative bacteria. ACS Infect. Dis. 7, 2073–2080. doi: 10.1021/acsinfecdis.1c00263, PMID: 34291902

[ref62] Wiegand I. Hilpert K. Hancock R. E. (2008). Agar and broth dilution methods to determine the minimal inhibitory concentration (MIC) of antimicrobial substances. Nat. Protoc. 3, 163–175. doi: 10.1038/nprot.2007.521, PMID: 18274517

[ref63] World Health Organization (2017). Antibacterial agents in clinical development: an analysis of the antibacterial clinical development pipeline, including tuberculosis. Geneva: WHO.

[ref64] Xie C. Yan J. Cao S. Liu R. Sun B. Xie Y.. (2022). Bi-layered disulfiram-loaded fiber membranes with antibacterial properties for wound dressing. Appl. Biochem. Biotechnol. 194, 1359–1372. doi: 10.1007/s12010-021-03663-0, PMID: 34714499

[ref65] Xu L. Tong J. Wu Y. Zhao S. Lin B. L. (2021). A computational evaluation of targeted oxidation strategy (TOS) for potential inhibition of SARS-CoV-2 by disulfiram and analogues. Biophys. Chem. 276:106610. doi: 10.1016/j.bpc.2021.106610, PMID: 34089978 PMC8161800

[ref66] Zheng Y. He L. Asiamah T. K. Otto M. (2018). Colonization of medical devices by staphylococci. Environ. Microbiol. 20, 3141–3153. doi: 10.1111/1462-2920.14129, PMID: 29633455 PMC6162163

