# Supplementary figures and images for: The combination of diethyldithiocarbamate and copper ions is active against Staphylococcus aureus and Staphylococcus epidermidis biofilms in vitro and in vivo
Source: Front Microbiol. 2022 Sep 9;13:999893. doi: 10.3389/fmicb.2022.999893 (PMC9500474; doi:10.3389/fmicb.2022.999893)

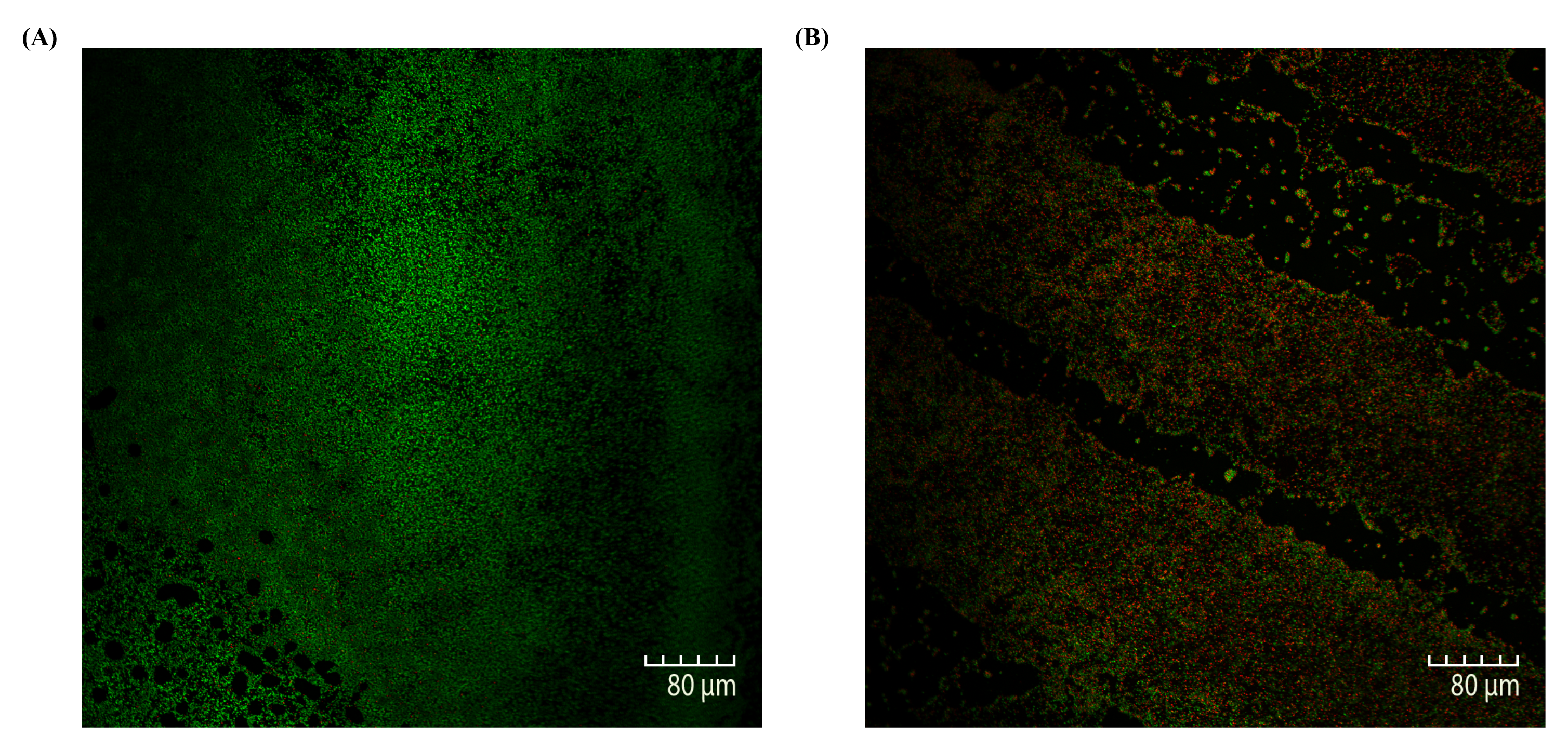

Supplement: Supplementary Figure 1 — Confocal microscopy images of stained MRSA Mu50 biofilms with LIVE/DEAD BacLight staining after treatment with 8 μg/ml diethyldithiocarbamate and 32 μg/ml Cu2+. Confocal microscopy images results: green = viable bacteria; red = dead bacteria. (A) Untreated MRSA Mu50 biofilm at 20 ×. (B) MRSA Mu50 biofilm after treatment with DDC-Cu2+ at 20 ×. [file Image_1.TIF]
